# Effects of a high-dose 24-h infusion of tranexamic acid on death and thromboembolic events in patients with acute gastrointestinal bleeding (HALT-IT): an international randomised, double-blind, placebo-controlled trial

**DOI:** 10.1016/S0140-6736(20)30848-5

**Published:** 2020-06-20

**Authors:** The HALT-IT Trial Collaborators, Ian Roberts, Haleema Shakur-Still, Adefemi Afolabi, Adegboyega Akere, Monica Arribas, Amy Brenner, Rizwana Chaudhri, Ian Gilmore, Kenneth Halligan, Irshad Hussain, Vipul Jairath, Kiran Javaid, Aasia Kayani, Ton Lisman, Raoul Mansukhani, Muttiullah Mutti, Muhammad Arif Nadeem, Richard Pollok, Jonathan Simmons, Majid Soomro, Simon Stanworth, Andrew Veitch, Christopher Hawkey, Adefemi Afolabi, Jack Cuzick, Kenneth Halligan, David Henry, Chris Metcalfe, Ian Roberts, Richard Gray, Alan Barkun, Suresh David, Philip Devereaux, Tony Brady, Ian Roberts, Haleema Shakur-Still, Timothy Coats, Phil Edwards, Ian Gilmore, Vipul Jairath, Katharine Ker, Daniela Manno, Simon Stanworth, Andrew Veitch, Monica Arribas, Emma Austin, Kiran Bal, Eni Balogun, Collette Barrow, Danielle Beaumont, Myriam Benyahia, Amy Brenner, Imogen Brooks, Madeleine Cargill, Laura Carrington, Phil Edwards, Lauren Frimley, Amber Geer, Daniel Gilbert, Catherine Gilliam, Julio Gil Onandia, Nayia Golfi, Daniel Hetherington, Courtenay Howe, Carolyn Hughes, David I'anson, Rob Jackson, Miland Joshi, Sneha Kansagra, Taemi Kawahara, Katharine Ker, Sergey Kostrov, Daniela Manno, Raoul Mansukhani, Hakim Miah, Bernard Ndungu, Kelly Needham, Aroudra Outtandy, Daniel Pearson, Tracey Pepple, Danielle Prowse, Nigel Quashi, Anna Quinn, Maria Ramos, Laura Ranopa, Mia Reid, Ian Roberts, Chris Roukas, Haleema Shakur-Still, Chelci Squires, Jemma Tanner, Andrew Thayne, Ruhama Uddin, Rizwana Chaudhri, Muttiullah Mutti, Kiran Javaid, Aasia Kayani, Bukola Fawole, Folasade Adenike Bello, Oladapo Olayemi, Adefemi Afolabi, Olujide Okunade, Olusade Adetayo, Rizwana Chaudhri, Muttiullah Mutti, Adefemi Afolabi, Folasade Adenike Bello, Bukola Fawole, Oladapo Olayemi, Hussein Khamis, Mohammad Shukri Bin Jahit, Tamar Gogichaishvili, Radu Bogdan Mateescu, Ajay Adhikaree, Abdelmounem Eltayeib Abdo, Mohammad Zaher, Conor Deasy, Joaquin Alvarez Gregori, Bobby Wellsh, Luke Lawton, Raghavendra Kamath, Adrian Barry, Racquel Carpio, Kay Finney, Holly Maguire, Martin James, Frank Coffey, Chris Gough, Lisa Sawers, Aye-Aye Thi, Jonathan Simmons, Claire Burnett, Nicola Jacques, Victoria Murray, Richard Pollok, Heather Jarman, Christine Lambe, Sarah Rounding, Simon Tucker, Romaih Al-Idari, Samuel Guest, Emma Stoddard, David Yeo, Colin Bergin, Elaine Hardy, Joanne Thunder, Paul Jhalli, Edward Hartley, Catherine Jarvis, Carly Swann, Matthew Reed, Bernadette Gallagher, Julia Grahamslaw, Rachel O'Brien, Timothy Harris, Geoffrey Bellhouse, Olivia Boulton, Imogen Skene, Adrian Stanley, Janet Johnstone, Donogh Maguire, Susan Thornton, Matthew Banks, Georgia Bercades, Daniel Marks, Jung Ryu, Timothy Harris, Claire Dowty, Jason Pott, Imogen Skene, James East, Adam Bailey, Sally Beer, Sian Davies, Andrew Appelboam, Daisy Mackle, Jennifer Small, Christiane Vorwerk, Rachel Atkins, Isobel Bradbury, Timothy Coats, Catriona Bryceland, Lisa McClelland, Martin Thomas, Kate Clayton, Angiy Michael, Stephen Haig, Saif Al-Nahhas, Tim Godfrey, Philip Boger, Rachel Comer, Barbara Watkins, Ola Afolabi, Shazad Afzal, Amanda Cowton, Simon Everett, Ruth Fazakerley, Felicia Onoviran, Jonathon Snook, Jackie Berry, Diane Simpson, Jeff Keep, Hannah Cotton, Sinead Helyar, Matthew Rutter, Tracey Johnston, Laura O'Rourke, Louisa Chan, Joanna Tambellini, Dawn Trodd, James Shutt, Sarah Moreton, Abby Oglesby, Adrian Boyle, Nicola Haeger, Susie Hardwick, Jason Kendall, Beverley Faulkner, Ruth Worner, Sarah Hearnshaw, Mary Doona, Maria Price, Laura Hunter, Maggie Bell, Vania Loureiro, Anthony Kehoe, Alison Jefferey, Rosalyn Squire, David Hartin, Stephanie Bell, Alexandra Newman, James Gagg, Jayne Foot, Sue Wakeford, Gabrielle May, Thomas Bartram, Paul Cumpstay, Lucy Parker, Rita Das, Sheik Pahary, Gavin Wright, Georgina Butt, Natasha Christmas, Sarah Wilson, Mohammed Ashfaq, Louise Chandler, Saif Al-Nahhas, Carrie Demetriou, Philip Kaye, Simon Carley, Andrew Brown, Lucy Jones, Amanda Whileman, John Greenaway, Julie Tregonning, Timothy Harris, Geoffrey Bellhouse, Avril Kuhrt, Steve Goodacre, John Jones, Charlotte Owen, Anu Mitra, Abby Harper-Payne, Nigel Trudgill, Anne Hayes, Faheem Butt, Gayle Clifford, Andrew Kinnon, Susan Fowler, Kris Pillay, Shweta Gidwani, Alistair McNair, Omer Omer, Tanya de Weymarn, Adnan Amin, Louisa Chan, Jane Martin, Nick Mathieu, Simon Barnes, James Turvill, Helen Sweeting, Morten Draegebo, Marion McNaught, Mandy Grocutt, Jordi Margalef, Julian Humphrey, Richard Jackson, Fionn Bellis, Jane Hunt, Anu Mitra, Alastair Stevenson, Nicholas Watson, Steven Barden, Stuart Paterson, Andrew Veitch, Chris Macdonald, David Hobday, Olu Orugun, Andrew Allison, Tristan Dyer, Samuel McBride, Wojciech Sawicki, Ben Rayner, Lynsey Flowerdew, Jamie Barbour, Jason Klein, Stephen Hood, Nicola Palmer, Jacob de Wolff, Achuth Shenoy, Nigel Trudgill, Peter Swallow, Rajaventhan Srirajaskanthan, Irshad Hussain, Hamza Arshad, Naeem Aslam, Anam Bangash, Muhammad Qamar, Haroon Zahoor, Muttiullah Mutti, Saba Arshad, Quratul ain Ghalib, Tehseen Hameed, Tayyaba Saif, Wajahat Shafi, Muhammad Arif Nadeem, Abid Ali, Shehroze Khan, Muhammad Muaaz, Ahmad Taj, Aamir Ghafoor, Aamir Afridi, Mansoor Ahmad, Mujahid Aslam, Sandeep Kumar, Majid Soomro, Mohsin Ali, Ubedullah Bughio, Adil Chang, Sana Shaikh, Syed Ahmad, Zeeshan Ali, Marium Waqar, Aiman Mushir, Sadaf Sattar, Saifullah Goraya, Sharmeen Aslam, Nighat Fatima, Saadia Noreen, Sheraz Saleem, Fazal Rahman, Nadeem Iqbal, Mohammad Khalid, Umar Riaz, Muhammad Umar, Tayyab Akhter, Javaria Khan, Noureen Misbah, Muhammad Afzal, Mobeen Kayani, Syed Shah, Shahida Tarar, Sherbat Khan, Yasir Iqbal, Essa Khan, Maqbool Reki, Tanveer Hussain, Shafqat Iqbal, Muhammad Khurram, Muhammad Shafi, Abrar Shaikh, Aijaz Ahmed, Ameet Kumar, Pinkey Sachdev, Khalid Mahmood Nasir, Zafar Iqbal Chaudhry, Muhammad Zubair, Ghias Tayyab, Junaid Mushtaq, Muhammad Nasir, Amir Khan, Amjad Ali, Sajjad Ali, Wasim Uddin, Sohaib Ahmed, Tazaeen Kazmi, Saleh Channa, Adeeqa Aman, Mouzam Shaikh, Tahir Rizvi, Amjad Hussain, Haider Zaigham Baqai, Zakawat Rasheed, Abdus Khan, Adeela Irfan, Aamir Husain, Asifa Aslam, Khalid Yahya, Salman Azhar, Mansoor Ul Haq, Adeel Afzal, Muhammad Imran, Iram Saeed, Aasim Yusuf, Mariam Hassan, Mumtaz Marwat, Muhammad Ishfaq, Tahir Bashir, Santosh Kumar, Sajjad Yaqoob, Abdul Wahid, Adegboyega Akere, Tinuola Fakoya, Temitope Oke, Edries Tejan, Oluwole Olaomi, Olawale Badejo, Okafor Nnaemaka, Nancy Ukwu, Olukayode Arowolo, Adewale Aderounmu, Funmilola Wuraola, Rose Ugiagbe, Alexander Atiri, Enadeghe Eghaghe, Adeleke Adekoya, Adedayo Oluyomi Tade, Olatunji Shonoiki, Samuel Olatoke, Toafiq Raji, Christopher Ekwunife, Chigozirim Onyekpere, Adamu Ahmed, Daniyan Muhammad, Emuobor Odeghe, Olufunmilayo Lesi, Azeberoje Osueni, Adamu Samaila, Aminu Nahuche, Akande Ajayi, Andrew Dongo, Uchenna Ijoma, Ademola Tolulope Adebanjo, Rufina Igetei, Monday Yilkudi, Kehinde Osisanya, Edith Nonyelum Okeke, Oguamanam Okezie Enwere, Serag Esmat, Omar Ashoush, Mazen Naga, Fady Nagy, Mostafa Saiid, Ahmed Shaker, Hussein Khamis, Ashraf Helmy, Saafan Saafan, Mohammed Abdel Monem, Jiffre Din, Khairul Azis, Muhyuddin Brukan, Sanjay Singh, Andee Zakaria, Shaik Farid, Nizam Hashim, Masykurin Mafauzy, Wan Najmi, Nil Amri, Xin Yi, Mohammad Hisyam, Elaine Ng, Zuhrirahimi Ramli, Shyang Yee Lim, Kelvin Voon, Sir Young Yam, Mohammad Jahit, Lee Joon, Besik Melikidze, Davit Kazaishvili, Tamar Gogichaishvili, Nino Grubelashvili, Baadur Mosidze, Gia Tomadze, Avto Megreladze, Ruxandra Oprita, Dorina Pestroiu Calescu, Camelia Chioncel, Andrei Ragea, Bogdan Mateescu, Bogdan Busuioc, Andrei Voiosu, Adrian Cotirlet, Iulia Pintilie, Mariana Jinga, Daniel Balaban, Marcel Tanău, Lucian Negreanu, Simona Bataga, Khushboo Priya, Shankar Baral, Anuj K.C., Vijay Sah, Vijay Yadav, Ajay Adhikaree, Abdelmounem Abdo, Dalia Ahmed, Marzouqah Al Anazi, Areej Al Balkhi, Conor Deasy, Joaquín Álvarez Gregori, Helio Fornieles Pérez, Arben Beqiri, Bobby Wellsh, Luke Lawton

## Abstract

**Background:**

Tranexamic acid reduces surgical bleeding and reduces death due to bleeding in patients with trauma. Meta-analyses of small trials show that tranexamic acid might decrease deaths from gastrointestinal bleeding. We aimed to assess the effects of tranexamic acid in patients with gastrointestinal bleeding.

**Methods:**

We did an international, multicentre, randomised, placebo-controlled trial in 164 hospitals in 15 countries. Patients were enrolled if the responsible clinician was uncertain whether to use tranexamic acid, were aged above the minimum age considered an adult in their country (either aged 16 years and older or aged 18 years and older), and had significant (defined as at risk of bleeding to death) upper or lower gastrointestinal bleeding. Patients were randomly assigned by selection of a numbered treatment pack from a box containing eight packs that were identical apart from the pack number. Patients received either a loading dose of 1 g tranexamic acid, which was added to 100 mL infusion bag of 0·9% sodium chloride and infused by slow intravenous injection over 10 min, followed by a maintenance dose of 3 g tranexamic acid added to 1 L of any isotonic intravenous solution and infused at 125 mg/h for 24 h, or placebo (sodium chloride 0·9%). Patients, caregivers, and those assessing outcomes were masked to allocation. The primary outcome was death due to bleeding within 5 days of randomisation; analysis excluded patients who received neither dose of the allocated treatment and those for whom outcome data on death were unavailable. This trial was registered with Current Controlled Trials, ISRCTN11225767, and ClinicalTrials.gov, NCT01658124.

**Findings:**

Between July 4, 2013, and June 21, 2019, we randomly allocated 12 009 patients to receive tranexamic acid (5994, 49·9%) or matching placebo (6015, 50·1%), of whom 11 952 (99·5%) received the first dose of the allocated treatment. Death due to bleeding within 5 days of randomisation occurred in 222 (4%) of 5956 patients in the tranexamic acid group and in 226 (4%) of 5981 patients in the placebo group (risk ratio [RR] 0·99, 95% CI 0·82–1·18). Arterial thromboembolic events (myocardial infarction or stroke) were similar in the tranexamic acid group and placebo group (42 [0·7%] of 5952 *vs* 46 [0·8%] of 5977; 0·92; 0·60 to 1·39). Venous thromboembolic events (deep vein thrombosis or pulmonary embolism) were higher in tranexamic acid group than in the placebo group (48 [0·8%] of 5952 *vs* 26 [0·4%] of 5977; RR 1·85; 95% CI 1·15 to 2·98).

**Interpretation:**

We found that tranexamic acid did not reduce death from gastrointestinal bleeding. On the basis of our results, tranexamic acid should not be used for the treatment of gastrointestinal bleeding outside the context of a randomised trial.

**Funding:**

UK National Institute for Health Research Health Technology Assessment Programme.

## Introduction

Acute severe gastrointestinal bleeding is a common cause of death worldwide.^1^ Bleeding can occur from the upper or lower gastrointestinal tract, but upper gastrointestinal bleeding is more common. The leading causes are peptic ulcer, oesophageal varices, and malignancy. The case fatality rate is approximately 10% for upper gastrointestinal bleeding and 3% for lower gastrointestinal bleeding.^2^, ^3^ Many patients re-bleed after initial haemostasis and those that do have a four-times increased risk of death.^4^ Patients with acute severe gastrointestinal bleeding usually present with haematemesis or melaena. Patients are often haemodynamically unstable and in need of urgent resuscitation. Acute management of gastrointestinal bleeding includes blood product transfusion, medical or endoscopic therapy, and surgery.

Tranexamic acid reduces bleeding by inhibiting blood clot breakdown (fibrinolysis). Tranexamic acid decreases surgical bleeding and reduces death due to bleeding in patients with traumatic and postpartum haemorrhage.^5^, ^6^, ^7^, ^8^ A systematic review and meta-analysis of randomised trials of tranexamic acid for upper gastrointestinal bleeding included seven trials with a total of 1654 patients.^9^ There was a large reduction in all-cause mortality with tranexamic acid (risk ratio [RR] 0·61, 95% CI 0·42–0·89; p=0·01). However, meta-analyses of small trials are prone to publication and other selection biases, and have a low positive predictive value when compared with results from large multicentre trials.^10^ Furthermore, even in aggregate, the trials included in the meta-analysis were too small to assess the effect of tranexamic acid on thromboembolic adverse events.^9^ Our objective was to quantify the effects of tranexamic acid on death and thromboembolic events in acute gastrointestinal bleeding.

**Research in context**

**Evidence before this study**

Before this study a Cochrane systematic review and meta-analysis of randomised trials of tranexamic acid for upper gastrointestinal bleeding included seven trials with a total of 1654 patients. There was a large reduction in mortality with tranexamic acid (pooled risk ratio [RR] 0·61, 95% CI 0·42–0·89; p=0·01). However, given the small size of the included trials and the potential for selection and other biases, we considered this evidence to be hypothesis generating, requiring confirmation in larger trials. Furthermore, there was substantial uncertainty about the risk of thromboembolic events with tranexamic acid (pooled RR 1·86, 95% CI 0·66–5·24).

**Added value of this study**

The HALT-IT trial included 12 009 patients from 164 hospitals in 15 countries. Adult patients with significant upper or lower gastrointestinal bleeding were randomly assigned to receive tranexamic acid (1 g loading dose followed by 3 g maintenance dose over 24 h) or matching placebo. Tranexamic acid did not reduce death from gastrointestinal bleeding (RR 0·99, 95% CI 0·82–1·18) but was associated with an increased risk of venous thromboembolic events (1·85, 1·15–2·98) and seizures (1·73, 1·03–2·93).

**Implications of all the available evidence**

The most recent update of the Cochrane review included eight small randomised trials with 1701 participants and showed a reduction in mortality with tranexamic acid (RR 0·60, 95% CI 0·42–0·87). Although we cannot entirely rule out a modest increase or decrease in death due to bleeding with tranexamic acid, we can rule out the large mortality reduction suggested by the Cochrane review. Furthermore, tranexamic acid appears to increase the risk of venous thromboembolic events in patients with gastrointestinal bleeding. On the basis of our results, tranexamic acid should not be used for the treatment of gastrointestinal bleeding outside the context of a randomised trial. Our results highlight the unreliability of meta-analyses of small trials.

## Methods

### Study design and participants

The HALT-IT trial is an international, randomised, double blind (participants and trial staff), placebo-controlled trial done in 164 hospitals in 15 countries (UK, Pakistan, Nigeria, Egypt, Malaysia, Georgia, Romania, Nepal, Sudan, Saudi Arabia, Spain, Ireland, Albania, Papua New Guinea, and Australia).^11^ Patients were enrolled if they were aged above the minimum age considered an adult in their country (either aged 16 years and older or aged 18 years and older) and if the responsible clinician was substantially uncertain whether to use tranexamic acid. The diagnosis of significant bleeding was clinical and significant was defined as a risk of bleeding to death and included patients with hypotension, tachycardia, or signs of shock, or those likely to need transfusion or urgent endoscopy or surgery.

Severe gastrointestinal bleeding is a frightening experience and blood loss can impact on a patient's mental and emotional state, impairing their decision making—the consent procedures considered this, as well as the need to treat urgently. If the patient was fully competent, written consent was sought. If capacity was impaired and a personal or professional representative was available, consent was sought from the representative. If neither were able to provide consent, it was waived and the patient was informed about the trial and consented for ongoing data collection as soon as possible afterwards. The trial was approved by the UK NRES Committee East of England (reference number 12/EE/0038), and by the national and local research ethics committees in all participating non-UK countries.

### Randomisation and masking

An independent statistician from Sealed Envelope (London, UK) generated randomisation numbers and these were given to Sharp Clinical Services UK (Crickhowell, UK), a Good Manufacturing Practice certified clinical trial service provider, to make treatment packs. When a patient was enrolled, the lowest numbered treatment pack was taken from a box of eight packs. Sharp Clinical Services was responsible for masking, which involved removing the manufacturer's label and replacing it with the clinical trial label and randomisation number. Apart from the randomisation number, the pack label text was identical for tranexamic acid and placebo. Patients, caregivers, and those assessing outcomes were masked to allocation. We checked the coding by testing each batch of ampoules with high-performance liquid chromatography to determine the contents. Block randomisation was used but randomisation was not stratified.

### Procedures

Eligible patients were randomly assigned to get tranexamic acid or placebo as soon as possible and treatment was started immediately. A loading dose of 1 g tranexamic acid or placebo (sodium chloride 0·9%) was added to a 100 mL infusion bag of 0·9% sodium chloride and infused by slow intravenous injection over 10 min, followed by a maintenance dose of 3 g tranexamic acid or placebo added to 1 L of any isotonic intravenous solution and infused at 125 mg/h for 24 h. Every patient was assigned a uniquely numbered treatment pack, which contained eight ampoules of tranexamic acid 500 mg or placebo, one 100 mL bag of 0·9% sodium chloride (to use with the loading dose), two sterile 10 mL syringes and needles, stickers with the trial details and randomisation number (for attaching to infusion bags, forms, and the medical records), and instructions. Pfizer, Sandwich, UK (PL 00057/0952) manufactured the tranexamic acid and Torbay and South Devon NHS Foundation Trust (MIA [IMP] 13079) manufactured the sodium chloride 0·9% placebo. We provided information for patients and representatives, consent forms, and data collection forms. Stickers, instructions, leaflets, and forms were in local languages.

Once randomly assigned, we collected outcome data even if the treatment was not given. Outcome data were collected at death, discharge from the randomising hospital, or 28 days after randomisation, whichever occurred first. Trial investigators and their institutions provided direct access to the source data for trial-related monitoring, audits, and regulatory inspections. Monitoring was done according to the Sponsor's Standard Operating Procedure and the trial protocol. Formal inspections were carried out by the relevant Regulatory Agencies including the UK Medicines and Healthcare products Regulatory Agency, Irish Health Products Regulatory Authority, and Nigeria's National Agency for Food and Drug Administration and Control. Adherence to allocation sequence was monitored throughout the trial and any out of sequence pack use was automatically flagged in the trial database and the investigators were retrained.

### Outcomes

The primary outcome was death due to bleeding within 5 days of randomisation. Cause of death was assigned by local principal investigators who provided a narrative of events leading to death. These were reviewed by the chief investigator (masked to treatment allocation) and queried if more information was needed to confirm whether death was due to bleeding or another cause. Secondary outcomes were death due to bleeding within 24 h and within 28 days of randomisation, all-cause and cause-specific mortality at 28 days, rebleeding within 24 h, within 5 days, and within 28 days of randomisation, surgery or radiological intervention, blood product transfusion, thromboembolic events (deep vein thrombosis, pulmonary embolism, stroke, and myocardial infarction), seizures, other complications (including other significant cardiac event, sepsis, pneumonia, respiratory failure, renal failure, liver failure), days in an intensive care unit, and functional status. The diagnosis of rebleeding was made by the clinician based on established criteria. A diagnosis of thromboembolic events was made using strict definitions and diagnostic criteria, including a clinical assessment, diagnostic imaging, biomarker tests, and post-mortem examination. Seizures were diagnosed by clinical assessment. Functional status was measured with the Katz Index of Independence in Activities of Daily Living either at hospital discharge or in-hospital at 28 days.

### Statistical analysis

The sample size calculation was initially based on all-cause mortality as the primary outcome since we expected that most deaths would be due to bleeding.^11^ However, while the trial was underway, we observed that over half of all deaths were due to non-bleeding causes. Accumulating evidence from other large trials of tranexamic acid showed no apparent effect on non-bleeding deaths.^12^ Furthermore, patients received tranexamic acid (or placebo) only for their initial bleed and because tranexamic acid has a short half-life (approximately 2 h), it will be largely eliminated within 2 days. As such, we did not expect tranexamic acid to reduce deaths from rebleeding episodes many weeks after randomisation. The primary outcome was therefore changed to death due to bleeding within 5 days of randomisation on Nov 21, 2018. Based on the amended primary outcome, assuming a risk of death due to bleeding of 4%, a study with 12 000 patients has about 85% power (two-sided α of 5%) to detect a clinically important 25% relative reduction in death due to bleeding from 4% to 3%.

We published the statistical analysis plan before unblinding.^13^ The plan gave our reasons for amending the primary outcome measure and for increasing the sample size. The main analyses compared those allocated tranexamic acid with those allocated to placebo on a modified intention-to-treat basis, excluding patients who received neither dose of the allocated treatment and those for whom outcome data on death were unavailable. We present effect estimates (RRs) with a measure of precision (95% CI). The safety of participants was overseen by an independent data monitoring committee, which reviewed four non-masked interim analyses. We planned to report four subgroup analyses to examine the effects of tranexamic acid on the primary outcome stratified by the following baseline characteristics: time to treatment (≤3 h, >3 h), site of bleeding (upper *vs* lower gastrointestinal), suspected variceal bleeding and comorbid liver disease compared with other or unknown causes of bleeding, and by clinical Rockall score. We modelled an interaction between the treatment effect and time to treatment with time to treatment as a continuous variable. We did post-hoc subgroup analyses to examine the effects of tranexamic acid on the primary outcome stratified by World Bank country income level (high *vs* low and middle income), anticoagulant use, and systolic blood pressure.

This trial was registered with Current Controlled Trials, ISRCTN11225767, and ClinicalTrials.gov, NCT01658124.

### Role of the funding source

The funders had no role in study design, data collection, data analysis, data interpretation, or writing of the report. The corresponding authors had full access to all the data in the study and had final responsibility for the decision to submit for publication.

## Results

**Figure 1:**
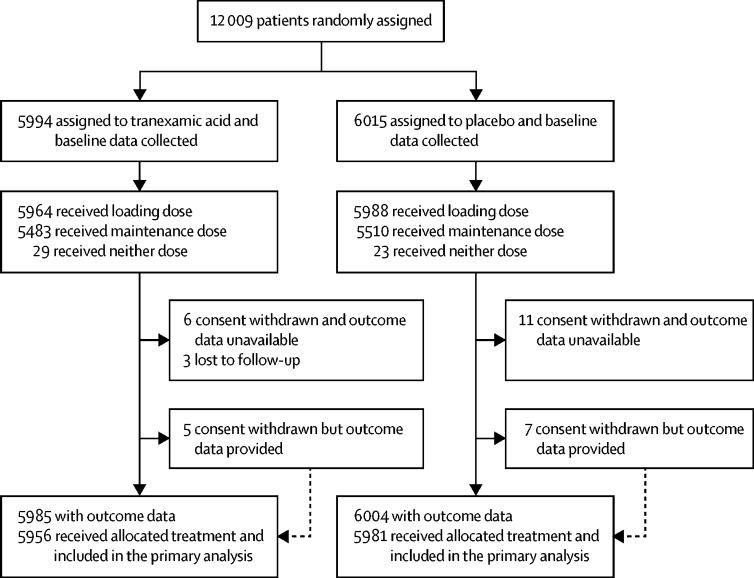
Trial profile

We enrolled the first patient on July 4, 2013, and the last on June 21, 2019. We stopped recruiting when the planned sample size was reached. When the decision to refine the primary outcome was made in Nov 21, 2018, we had recruited 10 190 patients. This decision was made blind to the accumulating trial data. 12 009 patients were enrolled and randomly assigned to receive either tranexamic acid (n=5994, 49·9%) or matching placebo (n=6015, 50·1%), of whom 11 952 (99·5%) received the first dose of the allocated treatment (figure 1). 29 patients (11 in the tranexamic acid group and 18 in the placebo group) withdrew consent after randomisation, but of those, 12 (five in the tranexamic acid group and seven in the placebo group) agreed to provide outcome data or had outcome data collected as part of adverse event reporting. We obtained primary outcome data for all but three patients in the tranexamic acid group. There were 14 protocol violations (seven in the tranexamic acid group and seven in the placebo group), 11 patients did not meet the inclusion criteria (ten received tranexamic acid before randomisation [six in the tranexamic acid group and four in the placebo group], one in the placebo group was younger than 16 years), and there were three consent protocol violations (one in the tranexamic acid group and two in the placebo group). A total of 28 patients were unmasked to treatment (17 patients because the hospital team wanted to administer tranexamic acid, six because of adverse events, three because of clinical concerns, and two as part of post-mortem investigations [13 in the tranexamic acid group and 15 in the placebo group]). 52 patients received neither dose of the allocated trial treatment (29 patients in the tranexamic acid group and 23 patients in the placebo group).

223 patients received antifibrinolytic drugs as part of their clinical care, outside of the trial protocol (105 patients in the tranexamic acid group and 118 patients in the placebo group).

**Figure 2:**
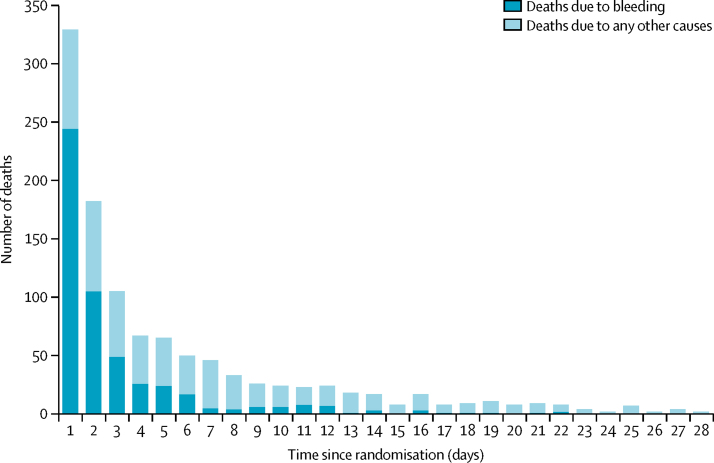
Mortality by days from randomisation

Baseline characteristics were similar between groups (table 1). Figure 2 shows the number of deaths and cause of death by days since randomisation. There were 1112 deaths. The median time to death was 55 h after randomisation (IQR 18·2–161·8).

**Table 1:** Baseline characteristics

	**Tranexamic acid (n=5994)**	**Placebo(n=6015)**
**Age at randomisation, years**		
Mean (SD)	58·1 (17·0)	58·1 (17·0)
<40	791 (13%)	779 (13%)
40–59	2356 (39%)	2333 (39%)
60–79	2078 (35%)	2130 (35%)
≥80	769 (13%)	773 (13%)
**Sex**		
Female	2142 (36%)	2124 (35%)
Male	3852 (64%)	3891 (65%)
**Time from onset to randomisation, h**		
Mean (SD)	21·4 (36·4)	22·5 (37·8)
≤3	960 (16%)	975 (16%)
>3–≤8	1607 (27%)	1551 (26%)
>8	3427 (57%)	3488 (58%)
Missing	0	1 (<1%)
**Suspected location of bleeding**		
Lower	674 (11%)	654 (11%)
Upper	5320 (89%)	5361 (89%)
**Haematemesis**		
Yes	4285 (72%)	4240 (71%)
No	1709 (29%)	1775 (30%)
**Melaena or fresh blood per rectum**		
Yes	4573 (76%)	4626 (77%)
No	1421 (24%)	1389 (23%)
**Suspected variceal bleeding**		
Yes	2694 (45%)	2739 (46%)
No	3300 (55%)	3276 (54%)
**Suspected active bleeding**		
Yes	5247 (88%)	5226 (87%)
No	747 (12%)	789 (13%)
**Systolic blood pressure, mm Hg**		
≥90	5222 (87%)	5216 (87%)
76–89	577 (10%)	577 (10%)
≤75	181 (3%)	201 (3%)
Missing	14 (<1%)	21 (<1%)
**Heart rate, beats per min**		
<77	812 (14%)	756 (13%)
77–91	1546 (26%)	1644 (27%)
92–107	1760 (29%)	1720 (29%)
>107	1864 (31%)	1885 (31%)
Missing	12 (<1%)	10 (<1%)
**Signs of shock**		
Yes	2574 (43%)	2648 (44%)
No	3420 (57%)	3367 (56%)
**Rockall score**		
1–2	1419 (24%)	1395 (23%)
3–4	2306 (38%)	2332 (39%)
5–7	2269 (38%)	2288 (38%)
**Taking anticoagulants**		
Yes	528 (9%)	500 (8%)
No	5422 (90%)	5466 (91%)
Unknown	44 (1%)	49 (1%)
**Emergency admission**		
Yes	5673 (95%)	5687 (94%)
No	321 (5%)	328 (6%)
**Major comorbidities**		
Cardiovascular	1108 (18%)	1132 (19%)
Respiratory	337 (6%)	324 (5%)
Liver	2432 (41%)	2532 (42%)
Renal	325 (5%)	310 (5%)
Malignancy	417 (7%)	382 (6%)
Other	999 (17%)	968 (16%)
Any comorbidity	4308 (72%)	4329 (72%)

Data are n (%) or mean (SD).

**Figure 3:**
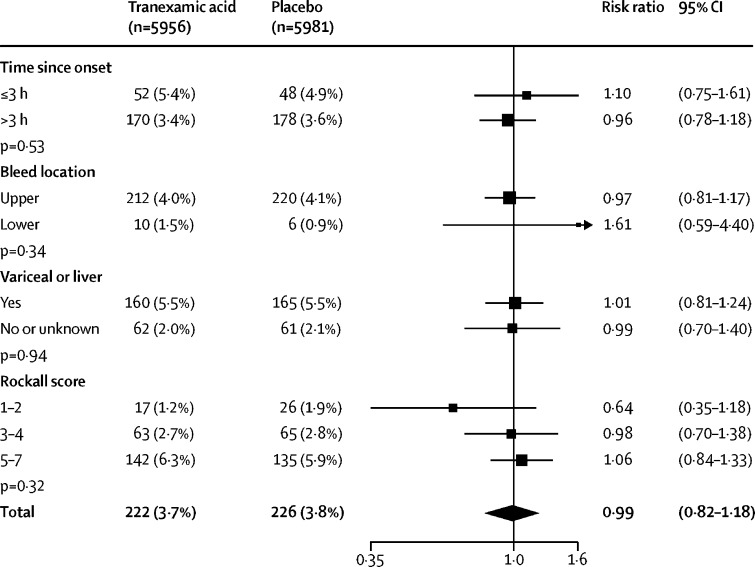
Effect of tranexamic acid on death due to bleeding within 5 days Analysis stratified by time since bleeding onset, suspected bleed location, suspected variceal bleeding or comorbid liver disease, and Rockall score.

Death due to bleeding within 5 days of randomisation (table 2) occurred in 222 (3·7%) of 5956 patients in the tranexamic acid group and in 226 (3·8%) of 5981 patients in the placebo group (RR 0·99, 95% CI 0·82–1·18). Similar results were obtained after adjusting for baseline covariates (0·98, 0·82–1·17) and in a per-protocol analysis (0·94, 0·71–1·23). When the 223 patients who received open-label antifibrinolytics were removed from the analysis, the results were similar (0·97, 0·81–1·17). We examined the effect of tranexamic acid on death due to bleeding within 5 days of randomisation in prespecified subgroup analyses stratified by time to treatment (heterogeneity p=0·53), location of bleeding (p=0·34), cause of bleeding (p=0·94), and clinical Rockall score (p=0·32) but recorded no evidence of heterogeneity for these factors (figure 3). When time since bleeding onset was modelled as a continuous variable there was no evidence of an interaction (heterogeneity p=0·53).

**Table 2:** Effect of tranexamic acid on death due to bleeding and rebleeding

	**Tranexamic acid (n=5956)**	**Placebo (n=5981)**	**Risk ratio (95% CI)**
Death due to bleeding within 24 h	124 (2·1%)	120 (2·0%)	1*·*04 (0*·*81–1*·*33)
Death due to bleeding within 5 days	222 (3·7%)	226 (3·8%)	0·99 (0·82–1·18)
Death due to bleeding within 28 days	253 (4·2%)	262 (4·4%)	0*·*97 (0*·*82–1*·*15)
Rebleeding within 24 h*	41 (0·7%)	41 (0·7%)	1*·*00 (0*·*65–1*·*55)
Rebleeding within 5 days*	287 (4·8%)	315 (5·3%)	0*·*91 (0*·*78–1*·*07)
Rebleeding within 28 days*	410 (6·8%)	448 (7·5%)	0*·*92 (0*·*81–1*·*05)

Data are n (%) and risk ratio (95% CI). Death or rebleeding in hospital during follow-up.

* Excludes 13 patients missing data on rebleed status or rebleed date.

We examined the effect of tranexamic acid on death due to bleeding within 5 days of randomisation stratified by World Bank country income level (high-income *vs* low-income and middle-income), anticoagulant use and systolic blood pressure. These exploratory analyses were not prespecified. The relative risks did not appear to vary by country income, anticoagulant use, or systolic blood pressure (appendix p 6).

Death due to bleeding within 24 h of randomisation occurred in 124 (2·1%) patients in the tranexamic acid group and 120 (2·0%) patients in the placebo group (RR 1·04, 95% CI 0·81–1·33). Death due to bleeding within 28 days of randomisation occurred in 253 (4·2%) patients in the tranexamic acid group and 262 (4·4%) patients in the placebo group (0·97, 0·82–1·15). Death from all-causes within 28 days of randomisation occurred in 564 patients (9·5%) in the tranexamic acid group and in 548 patients (9·2%) in the placebo group (1·03, 0·92–1·16; table 3). The proportion of patients with rebleeding was similar in both groups at 24 h, 5 days, and 28 days after randomisation (table 3). The proportion of patients who had surgery, radiological intervention, and blood product transfusion was also similar in both groups (table 4).

**Table 3:** Effect of tranexamic acid on all-cause death

	**Tranexamic acid (n=5956)**	**Placebo(n=5981)**	**Risk ratio(95% CI)**
Bleeding	253 (4*·2%*)	262 (4*·4%*)	0*·*97 (0*·*82–1*·*15)
Thromboembolic event	26 (0·4%)	17 (0·3%)	1*·*54 (0*·*83–2*·*83)
Organ failure	109 (1·8%)	114 (1·9%)	0*·*96 (0*·*74–1*·*25)
Pneumonia	57 (1*·0%*)	42 (0·7%)	1*·*36 (0*·*92–2*·*03)
Sepsis	33 (0·6%)	49 (0·8%)	0*·*68 (0*·*44–1*·*05)
Malignancy	65 (1*·1%*)	40 (0·7%)	1*·*63 (1*·*10–2*·*42)
Other	21 (0·4%)	24 (0·4%)	0*·*88 (0*·*49–1*·*58)
All cause	564 (9·5%)	548 (9*·2%*)	1*·*03 (0*·*92–1*·*16)

Data are n (%) and risk ratio (95% CI). Death in hospital during follow-up.

**Table 4:** Effect of tranexamic acid on the need for surgical, endoscopic, and radiological interventions or blood product transfusion

	**Tranexamic acid**	**Placebo**	**Outcomes**
**Interventions**			
Diagnostic endoscopy	4781/5953 (80·3%)	4729/5978 (79*·1%*)	1*·*02 (1*·*00 to 1*·*03)
Therapeutic endoscopy	2542/5952 (42·7%)	2658/5978 (44.5%)	0*·*96 (0*·*92 to 1*·*00)
Diagnostic radiological procedure	1704/5953 (28·6%)	1744/5978 (29*·2%*)	0*·*98 (0*·*93 to 1*·*04)
Therapeutic radiological procedure	74/5953 (1·2%)	89/5978(1·5%)	0*·*83 (0*·*61 to 1*·*13)
Surgical intervention	146/5953 (2·5%)	158/5978 (2·6%)	0*·*93 (0*·*74 to 1*·*16)
Any surgical, endoscopic, or radiological intervention	5216/5956 (87·6%)	5236/5981 (87·5%)	1*·*00 (0*·*99 to 1*·*01)
Any transfusion	4076/5951 (68·5%)	4129/5978 (69*·1%*)	0*·*99 (0*·*97 to 1*·*02)
Whole blood or red cells	3984/4076 (97·7%)	4018/4129 (97*·3%*)	1*·*00 (1*·*00 to 1*·*01)
Frozen plasma	910/4076 (22·3%)	993/4129 (24*·0%*)	0*·*93 (0*·*86 to 1*·*00)
Any platelets	219/4076 (5·4%)	255/4129 (6*·2%*)	0*·*87 (0*·*73 to 1*·*04)
**Blood product transfusions**			
Units of whole blood or red cells	2*·*8 (2*·*4)	2*·*9 (2*·*7)	−0·06 (0*·*05 to −0*·*18)
Units of frozen plasma	0*·*9 (2*·*4)	1*·*0 (2*·*6)	−0*·*05 (−0*·*01 to −0*·*23)
Units of any platelets	0*·*2 (0*·*9)	0*·*2 (1*·*0)	−0*·*02 (0*·*02 to −0*·*06)

Data for interventions are n/N (%) and risk ratio (95% CI); data for blood product transfusions are mean (SD) and difference in means (95% CI).

The risk of fatal or non-fatal thromboembolic events and arterial thromboembolic events (myocardial infarction or stroke) was similar in the tranexamic acid group and the placebo group (table 5). The risk of venous thromboembolic events (deep vein thrombosis or pulmonary embolus) was higher in the tranexamic group than in the placebo group (table 5) and similar risk was observed after excluding patients who did not receive the maintenance dose (42 events with tranexamic acid *vs* 20 with placebo); RR 2·11, 95% CI 1·24–3·59). In an exploratory subgroup analysis, the risk of venous thromboembolic events was higher in patients with suspected variceal bleeding or liver disease (14 *vs* two events; 7·26, 1·65–31·90) than in patients with other causes of bleeding (34 *vs* 24 events; 1·38, 0·82–2·32; p=0·035 for heterogeneity). The risk of renal, hepatic, and respiratory failure, cardiac events, sepsis, and pneumonia was similar in tranexamic acid and placebo treated patients (table 5). Seizures occurred in 38 patients on tranexamic acid and 22 on placebo (0·6% *vs* 0·4%; 1·73, 1·03–2·93; table 5), and after excluding patients who did not receive the maintenance dose the corresponding numbers were 33 versus 17 events (1·95, 1·09–3·50).

**Table 5:** Complications and self-care capacity in study groups

	**Tranexamic acid**	**Placebo**	**Outcomes**
**Complications**			
Any thromboembolic event	86/5952 (1·4%)	72/5977 (1·2%)	1*·*20 (0*·*88 to 1*·*64)
Venous events (deep vein thrombosis, pulmonary embolism)	48/5952 (0·8%)	26/5977 (0·4%)	1*·*85 (1*·*15 to 2*·*98)
Deep vein thrombosis	23/5952 (0·4%)	12/5977 (0·2%)	1*·*92 (0*·*96 to 3*·*86)
Pulmonary embolism	28/5952 (0·5%)	16/5977 (0·3%)	1*·*76 (0*·*95 to 3*·*24)
Arterial events (myocardial infarction, stroke)	42/5952 (0·7%)	46/5977 (0·8%)	0*·*92 (0*·*60 to 1*·*39)
Myocardial infarction	24/5952 (0·4%)	28/5977 (0·5%)	0*·*86 (0*·*50 to 1*·*48)
Stroke	19/5952 (0·3%)	18/5977 (0·3%)	1*·*06 (0*·*56 to 2*·*02)
Renal failure	142/5951 (2·4%)	157/5978 (2·6%)	0*·*91 (0*·*73 to 1*·*14)
Liver failure	196/5952 (3·3%)	184/5977 (3·1%)	1*·*07 (0*·*88 to 1*·*30)
Respiratory failure	105/5952 (1·8%)	131/5978 (2·2%)	0*·*81 (0*·*62 to 1*·*04)
Cardiac event	100/5952 (1·7%)	89/5977 (1·5%)	1*·*13 (0*·*85 to 1*·*50)
Sepsis	210/5952 (3·5%)	216/5977 (3·6%)	0*·*98 (0*·*81 to 1*·*18)
Pneumonia	193/5952 (3·2%)	174/5978 (2·9%)	1*·*11 (0*·*91 to 1*·*36)
Seizure	38/5952 (0·6%)	22/5977 (0·4%)	1*·*73 (1*·*03 to 2*·*93)
**Self-care capacity**			
Days in ICU	0*·*4 (1*·*8)	0*·*4 (2*·*0)	−0*·*06 (0*·*01 to −0*·*13)
Katz score	5*·*5 (1*·*5)	5*·*5 (1*·*4)	−0*·*03 (0*·*02 to −0*·*09)

Data for complications are n/N (%) and risk ratio (95% CI); data for self-care capacity are mean (SD) and difference in means (95% CI). Thromboembolic events and complications are not mutually exclusive. ICU=intensive care unit.

The mean number of days spent in intensive care was similar in both groups (table 5). The mean score on the Katz Index of Independence in Activities of Daily Living was also similar in both groups (table 5).

## Discussion

In this trial, tranexamic acid did not reduce death from gastrointestinal bleeding but was associated with an increased risk of venous thromboembolic events and seizures. The proportion of patients with rebleeding was similar in the tranexamic acid and placebo groups.

The randomisation method ensured that participating clinicians had no foreknowledge of the treatment allocation and placebo control ensured outcome assessment was blind to treatment group. The inclusion criteria were clinical, reflecting the full range of gastrointestinal bleeding presentations that doctors face in day-to-day practice. Baseline prognostic factors were well balanced and almost all randomly assigned patients were followed up. The primary outcome was death due to bleeding within 5 days of randomisation. Our scientific reasons for pre-specifying death due to bleeding as the primary outcome in the statistical analysis plan are presented in detail elsewhere.^12^ Although some misclassification of cause of death is possible, the assessment was masked to the treatment group. However, because there was no evidence of a treatment effect for the prespecified primary endpoint (death due to bleeding at 5 days) or for death from any cause at 28 days, the choice of endpoint does not influence the interpretation of the results. Misclassification might also have affected our subgroup analyses because at the time of recruitment the site and cause of bleeding cannot be known with certainty. Our use of the pre-endoscopy Rockall score might have misclassified baseline risk.^14^ To minimise the risk of false positives, we used strict criteria to diagnose thromboembolic events, including a positive result on imaging (eg, ultrasound) or at post-mortem examination. Although using this criteria might have led to some under reporting, because the diagnostic tests have high specificity, the relative risk estimates should be unbiased. Although some patients received antifibrinolytics outside of the protocol, the treatment effect was the same when these patients were excluded. Although this is one of the largest randomised trials in gastrointestinal bleeding, we cannot rule out a modest increase or decrease in death due to bleeding with tranexamic acid. That said, we can rule out the large mortality reduction suggested by the Cochrane systematic review and meta-analysis of previous small trials.^9^

Administration of tranexamic acid within 3 h of bleeding onset reduces death due to bleeding in trauma and post-partum haemorrhage without increasing the risk of thromboembolic events. In these bleeding scenarios, the timing of onset is easy to determine, most patients present early, and there are well documented changes in fibrinolysis that provide a biological rationale for tranexamic acid treatment.^15^, ^16^ However, in gastrointestinal bleeding it is difficult to determine the time of bleeding onset, presentation is often delayed (over 80% of patients presented more than 3 h after bleeding onset), and the contribution of increased fibrinolysis to bleeding is less clear.

Almost half of the patients included in our trial had suspected variceal bleeding due to liver disease and because these patients had a greater risk of death, they accounted for nearly three-quarters of deaths. Recent research shows that acutely ill patients with cirrhosis have a mixed fibrinolytic phenotype.^17^ Some have increased fibrinolysis, but others have profound hypofibrinolysis. The prevalence of hypofibrinolysis appears to be greatest in the most critically ill patients. Using the same clot lysis assay, reduced fibrinolysis has been shown to be associated with a small increased risk of venous thrombosis.^18^ In our trial, the increased risk of venous thromboembolic events with tranexamic acid appeared to be more marked in patients with liver disease, although this was an exploratory subgroup analysis and there was no strong evidence for heterogeneity. Nevertheless, reduced fibrinolysis in patients with liver disease might explain the absence of reduction in bleeding deaths with tranexamic acid and the increased risk of venous thromboembolic events.

The dose of tranexamic acid used in this trial was higher and the duration of treatment was longer (4 g over 24 h) than in randomised trials of tranexamic acid in trauma (2 g over 8 h) or post-partum haemorrhage (1 g bolus with a repeat 1 g dose if bleeding continued), which did not record any increase in adverse events with tranexamic acid. Patients with gastrointestinal bleeding often rebleed after initial haemostasis, particularly within the first 24 h. Because tranexamic acid has a short half-life, we used a longer treatment duration to cover this high-risk period. Furthermore, previous trials in gastrointestinal bleeding that appeared to show a large mortality reduction with tranexamic acid used a high dose and a longer duration of treatment than trials in trauma and post-partum haemorrhage.^9^ The longer duration of tranexamic acid treatment in this trial might explain the increased risk of venous thromboembolic events and the higher dose could possibly explain the increased risk of seizures.^19^

In summary, we found no evidence that tranexamic acid decreases the risk of death in patients with gastrointestinal bleeding. Our results caution against a uniform approach to the management of patients with major haemorrhage and highlight the need for randomised trials targeted at specific pathophysiological processes. Because gastrointestinal bleeding is a licensed indication for tranexamic acid, our results could have regulatory implications. Although this trial can rule out the large mortality reduction suggested by the meta-analysis of previous small trials, it cannot rule out more modest treatment effects. Because tranexamic acid reduces bleeding deaths in patients with traumatic and postpartum haemorrhage, individual patient data meta-analyses should assess the strength of the evidence that the effectiveness and safety of tranexamic acid varies by the site and cause of bleeding.

Correspondence to: Clinical Trials Unit, London School of Hygiene & Tropical Medicine, London WC1 E7HT, UK haltit@lshtm.ac.uk

## Data sharing

Following publication of the primary and secondary analyses detailed in this statistical analysis plan, individual de-identified patient data, including data dictionary, will be made available via our data sharing portal, The Free Bank of Injury and Emergency Research Data (freeBIRD) website indefinitely. This will allow for maximum utilisation of the data to improve patient care and advance medical knowledge. The trial protocol, statistical analysis plan and trial publications will be freely available online.
