# Supplementary material for: Effects of a high-dose 24-h infusion of tranexamic acid on death and thromboembolic events in patients with acute gastrointestinal bleeding (HALT-IT): an international randomised, double-blind, placebo-controlled trial
Source: Lancet. 2020 Jun 20;395(10241):1927–36. doi: 10.1016/S0140-6736(20)30848-5 (PMC7306161; doi:10.1016/S0140-6736(20)30848-5)
Supplement: Supplementary appendix [file mmc1.pdf]

THE LANCET

Supplementary appendix

This appendix formed part of the original submission and has been peer reviewed. We post it as supplied by the authors.

Supplement to: The HALT-IT Trial Collaborators. Effects of a high-dose 24-h infusion of tranexamic acid on death and thromboembolic events in patients with acute gastrointestinal bleeding (HALT-IT): an international randomised, double-blind, placebo-controlled trial. *Lancet* 2020; **395:** 1927–36.

Supplementary web appendix

Table S1: Adverse events

	TXA	Placebo	Total
Abdominal distension	0	1	1
Abdominal pain	2	3	5
Abscess	1	1	2
Acute confusion state	3	1	4
Acute enterocolitis	1	0	1
Adenocarcinoma	0	1	1
Alcohol Intoxication	1	0	1
Alcoholic withdrawal symptoms	1	2	3
Allergic reaction	5	6	11
Anaemia	5	13	18
Angina pectoris	2	0	2
Angiodysplasia	1	0	1
Appendicitis	1	0	1
Arterioenteric fistula	1	0	1
Ascites	2	4	6
Aspiration	0	1	1
Atrial fibrillation	0	2	2
Bowel infarction	0	1	1
Bowel perforation	0	1	1
Brain lesion	0	1	1
COPD exacerbation	3	2	5
Calculus biliary	1	0	1
Cardiac arrest	0	1	1
Cardiac failure congestive	0	2	2
Cardiac failure congestive	0	1	1
Cellulitis	3	4	7
Cerebral infarction	1	0	1
Chemotherapy NOS	0	2	2
Chest pain	2	2	4
Cholangiocarcinoma	0	1	1
Cholangitis	1	0	1
Cholecystitis	0	1	1
Chronic venous insufficiency	0	1	1
Clostridium difficile infection	2	6	8
Coagulopathy	1	0	1
Colorectal cancer	0	1	1
Conjunctivitis	1	0	1
Constipation	5	3	8
Contusion	1	0	1
Crohn's disease	0	1	1
Crystal arthropathy	0	1	1
Cyanosis	0	1	1
Deep vein thrombosis	4	1	5
Dehydration	0	1	1
Delirium	1	2	3
Diarrhoea	3	2	5
Diverticular disease	3	1	4
Duodenal perforation	1	0	1
Dyspepsia	0	1	1
Embolus in foot	1	0	1
Encephalopathy	2	2	4
Enterocutaneous fistula	1	0	1
Epistaxis	1	1	2
Erythema	1	1	2
Fall	6	9	15

Febrile neutropenia	2	0	2
Fluid overload	1	1	2
Fluid retention	1	0	1
Fracture of unspecified intracapsular femur	0	1	1
Frailty	1	0	1
Gallbladder polyp	1	0	1
Gastric cancer	1	0	1
Gastric ulcer	1	0	1
Gastritis	1	4	5
Gastroenteritis	1	1	2
Gastrointestinal haemorrhage	66	79	145
Gastrointestinal haemorrhage	0	1	1
Gout	3	1	4
Groin pain	1	0	1
Haematoma	1	0	1
Haematuria traumatic	0	1	1
Haemodilution	1	0	1
Haemorrhoids	2	2	4
Head injury	1	0	1
Heart failure	0	1	1
Hemicolectomy	0	1	1
Hepatic cirrhosis	6	3	9
Hepatic encephalopathy	0	1	1
Hepatitis	0	1	1
Hepatocellular carcinoma	0	1	1
Hepatorenal syndrome	1	0	1
Humerus fracture	0	1	1
Hydrocele	0	1	1
Hypernatraemia	0	1	1
Hypoglycaemia	2	2	4
Hypokalaemia	2	1	3
Hypomagnesaemia	1	0	1
Hyponatraemia	0	1	1
Hyponatremia	1	1	2
Hypotension	1	0	1
Hypoxia	0	1	1
Infected skin ulcer	0	1	1
International normalized ratio abnormal	1	0	1
Intestinal stoma leak	0	1	1
Ischaemic heart disease	0	1	1
Ischaemic hepatitis	0	1	1
Ischaemic stroke	3	3	6
Jaundice	0	1	1
Large bowel obstruction	1	0	1
Leg ischaemia	0	1	1
Liver abscess	0	1	1
Liver carcinoma ruptured	1	0	1
Liver cirrhosis	1	0	1
Lower respiratory tract infection	1	1	2
Lung cancer	1	0	1
Mallory Weiss tear	0	1	1
Mastoid effusion	0	1	1
Meningioma	1	0	1
Multi organ failure	2	0	2
Myocardial infarction	2	1	3
Nasogastric tube irritation	1	0	1
Necrotising otitis externa	0	1	1
Neuropathy peripheral	0	1	1
Obstructive jaundice	0	1	1
Oedema	1	3	4
Oesophageal cancer	1	1	2

Oesophageal candida	0	3	3
Oesophagitis	0	3	3
Overdose	1	1	2
Pain	1	0	1
Palpitations	1	0	1
Pancreatitis	1	0	1
Panic attack	1	0	1
Perforated gastric ulcer	1	0	1
Pericardial effusion	1	0	1
Peritonitis	4	0	4
Pharyngitis	1	0	1
Phlebitis	2	0	2
Pleural effusion	0	2	2
Pneumonia	9	3	12
Portal vein thrombosis	1	1	2
Postural hypotension	1	2	3
Pressure sore	1	1	2
Pulmonary embolism	3	3	6
Pulmonary haemorrhage	1	0	1
Pulmonary oedema	1	0	1
Pyelonephritis	0	1	1
Pyrexia	3	3	6
Rash	1	0	1
Refeeding syndrome	0	2	2
Renal failure	2	1	3
Respiratory infection	12	1	13
Seizure	3	1	4
Sepsis	6	7	13
Shingles	0	1	1
Shortness of breath	1	0	1
Small bowel obstruction	2	2	4
Staphylococcal bacteremia	1	0	1
Stoma site bleeding	1	0	1
Suicidal ideation	0	1	1
Supraventricular tachycardia	0	1	1
Swollen lips	1	0	1
Syncope	1	1	2
Thoracic aneurysm, ruptured	0	1	1
Thrombocytopenia	1	1	2
Thrombosis	2	1	3
Transfusion reaction	0	1	1
Transient ischaemic attack	1	2	3
Type 2 diabetes mellitus	1	0	1
Upper gastrointestinal haemorrhage	1	0	1
Urinary incontinence	0	1	1
Urinary retention	0	1	1
Urinary retention	1	0	1
Urinary tract infection	11	10	21
Urinary tract infection	1	2	3
Ventricular tachycardia	1	0	1
Vomiting	1	2	3
**Total**	**273**	**281**	**554**

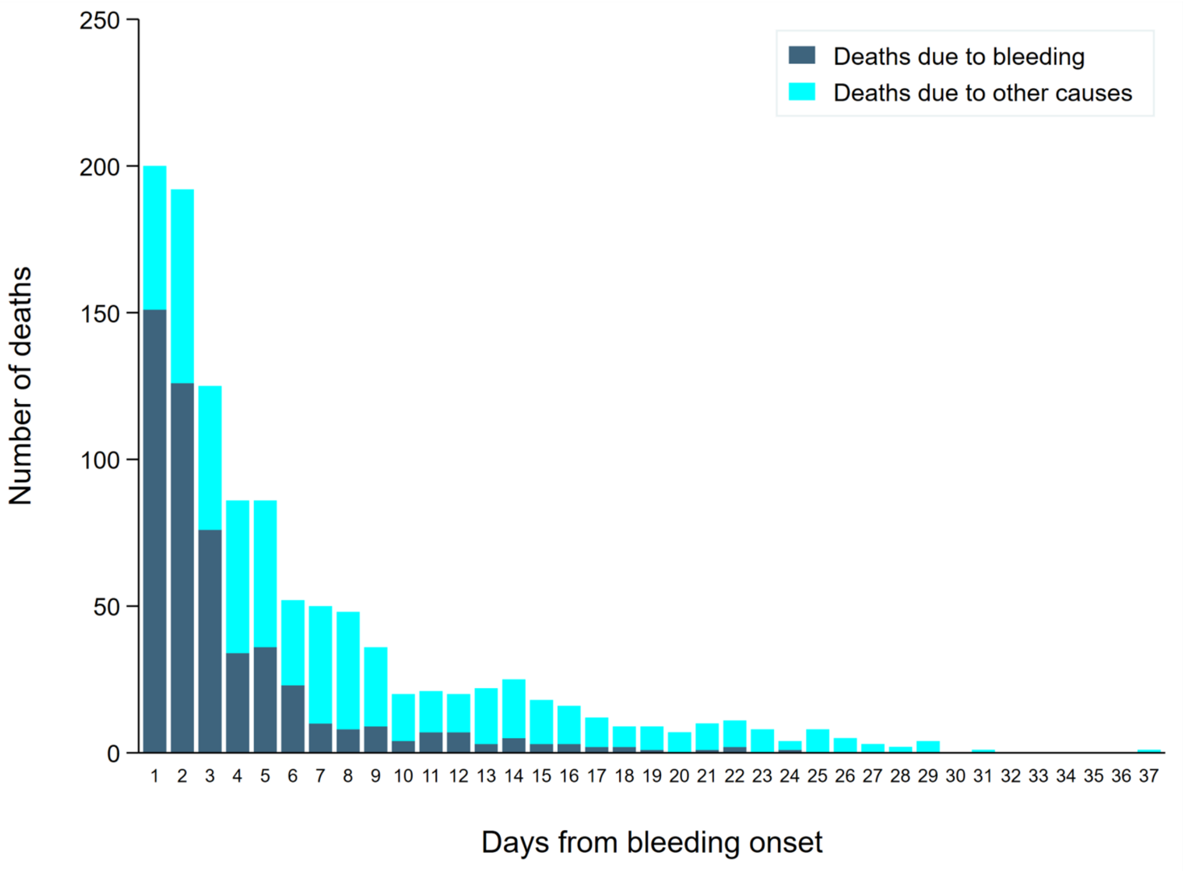

Figure S1. Mortality by days from bleeding onset

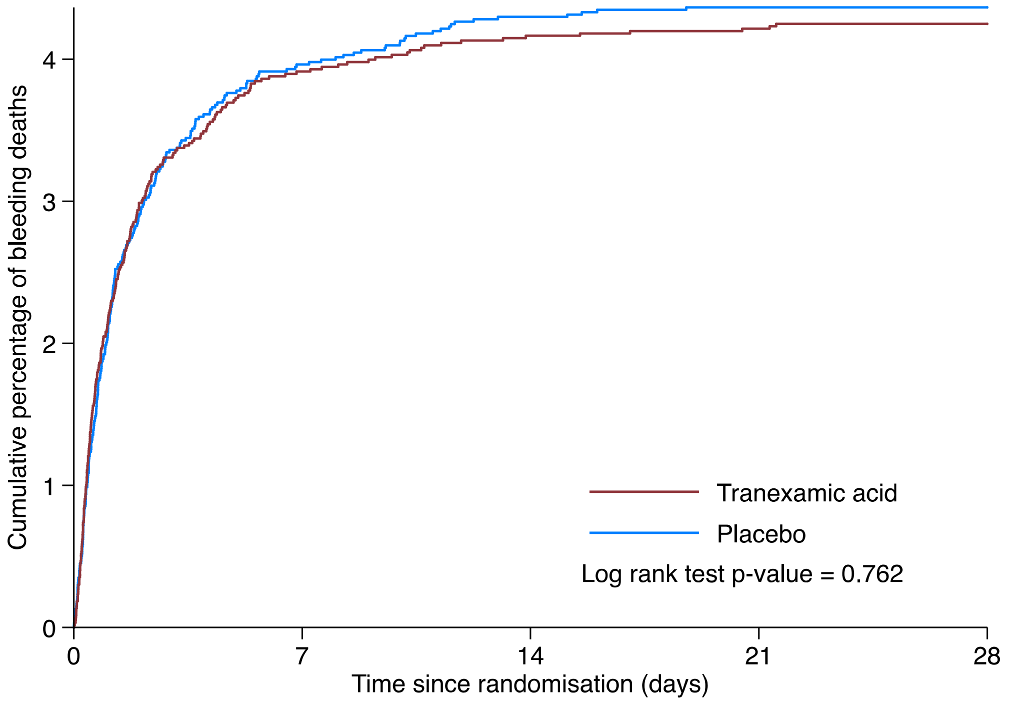

Number at risk

TXA	5956	5723	5708	5705	5703
Placebo	5981	5744	5724	5720	5720

Figure S2. Cumulative incidence plot of death due to bleeding. Hazard Ratio = 0.97, 95% CI 0.82-1.16
